# Hydroxychloroquine may reduce risk of Pneumocystis pneumonia in lupus patients: a Nationwide, population-based case-control study

**DOI:** 10.1186/s12879-020-4826-1

**Published:** 2020-02-10

**Authors:** Kai-Jieh Yeo, Hsin-Hua Chen, Yi-Ming Chen, Ching-Heng Lin, Der-Yuan Chen, Chih-Ming Lai, Wen-Cheng Chao

**Affiliations:** 1 Rheumatology and Immunology Center, China Medical University Hospital, Taichung, Taiwan; 2 Department of the Medical Research, Taichung Veterans General Hospital, Taichung, Taiwan; 3 Department of Internal Medicine, Taichung Veterans General Hospital, Taichung, Taiwan; 4 Institute of Biomedical Science and Rong-Hsing Research Center for Translational Medicine, Chung Hsing University, Taichung, Taiwan; 5 School of Medicine, National Yang-Ming University, Taipei, Taiwan; 6 Institute of Public Health and Community Medicine Research Center, National Yang-Ming University, Taipei, Taiwan; 7 Department of Industrial Engineering and Enterprise Information, Tung Hai University, Taichung, Taiwan; 8 Department of Healthcare Management, National Taipei University of Nursing and Health Sciences, Taipei, Taiwan; 9 Department of Public Health, College of Medicine, Fu Jen Catholic University, New Taipei City, Taiwan; 10 Translational Medicine Laboratory, Rheumatic Diseases Research Center, China Medical University Hospital, Taichung, Taiwan; 11 School of Medicine, China Medical University, Taichung, Taiwan; 12 Department of Critical Care Medicine, Taichung Veterans General Hospital, Taichung, Taiwan; 13 Department of Neurosurgery, Neurological Institute, Taichung Veterans General Hospital, Taichung, Taiwan; 14 Department of Business Administration, National Changhua University of Education, Changhua, Taiwan

**Keywords:** Lupus, Pneumocystis pneumonia, Hydroxychloroquine, Glucocorticoid, Mycophenolate mofetil

## Abstract

**Background:**

Pneumocystis pneumonia (PCP) is increasingly being diagnosed in patients with systemic lupus erythematosus (SLE), and hydroxychloroquine (HCQ) has been found to possess antifungal activities. We hence aimed to investigate the association between HCQ and PCP risk among patients with SLE.

**Methods:**

Using the 1997–2013 nationwide claim data, we identified 24,343 newly-diagnosed SLE patients. We then identified 58 PCP cases and selected 348 non-PCP controls matching (1:6) by age, sex, disease duration and the year of PCP diagnosis date. The risk of PCP was assessed by determing odds ratios (ORs) with 95% confidence intervals (CIs) by using multivariable conditional logistic regression.

**Results:**

The risk of PCP was associated with moderate to severe renal disease (OR 6.73, 95% CI 1.98–22.92), higher doses of glucocorticoids (≤5 mg/day, reference; 5–10 mg/day, OR 25.88, 95% CI 2.97–225.33; > 10 mg/day, OR 286.58, 95% CI 28.58–> 999), higher 3-month cumulative dose of cyclophosphamide (not use, reference; ≤1.4 g, OR 0.64, 95% CI 0.14–3.01; > 1.4 g, OR 11.52, 95% CI 1.97–67.39) and use of mycophenolate mofetil/mycophenolic acid (OR 50.79, 95% CI 5.32–484.77), whereas 3-month cumulative dose of HCQ was associated with a reduced risk of PCP among patients with SLE (not use, reference; ≤14 g, OR 0.69, 95% CI 0.21–2.24; > 14 g, OR 0.20, 95% CI 0.05–0.71).

**Conclusions:**

This study demonstrated incident PCP was associated with mycophenolate mofetil/mycophenolic acid use and higher doses of cyclophosphamide or glucocorticoid, whereas the use of a higher dose of HCQ was associated with a reduced risk of PCP in lupus patients.

## Background

Pneumocystis pneumonia (PCP), caused by *Pneumocystis jirovecii,* is a potentially life-threatening infection and is increasingly diagnosed in the immunocompromised patient without human immunodeficiency virus infection, including transplant recipients and the patient with malignancies or autoimmune diseases [1, 2]. Glucocorticoids (GC) and a number of immunosuppressants, mainly cyclophosphamide, and have been identified to be associated with an increased risk for PCP in patients with systemic lupus erythematosus (SLE) [3]. Notably, hydroxychloroquine (HCQ) and sulfasalazine (SSZ) have been found to have anti-fungal activities; therefore, it is crucial to address the risk for PCP in lupus patients receiving HCQ or SSZ [4–6]. One recently published Japanese multicenter study reported a reduced risk for PCP in patients with rheumatoid arthritis (RA) receiving SSZ [7]. HCQ is a frequently used medication in patients with SLE; therefore, there is a crucial need to determine the distinct risks for PCP among medications, including HCQ and immunosuppressants, in patients with SLE. In this study, we aimed to explore the individual risks for PCP of frequently administered medications including GC as well as immunosuppressants and to determine the potential protective effect of HCQ against PCP among patients with SLE by using a Taiwanes population-based claim database.

## Methods

### Ethical approval

The present study was approved by the Institutional Review Board of Taichung Veterans General Hospital, Taiwan (CE14149B-3). Written informed consent was waived due to that all of the individual data were de-identified.

### Study design

The present study was a retrospective case-control study.

### Data source

The Taiwanese single-payer National Health Insurance (NHI) program was launched in 1995 with wide coverage that approximately 99.5% of Taiwan’s population were enrolled in 2015 [8]. The National Health Insurance Research Database (NHIRD) is the database of the aforementioned NHI program containing all of the original claim profiles. In the present study, we used both the inpatient and ambulatory data from the 1997–2013 NHIRD to identify newly-diagnosed lupus patients from 2001 to 2013. Notably, in Taiwan, those with a certain major illness, so-called catastrophic illness, including cancer and a number of autoimmune diseases, including SLE, are exempted from co-payment. Furthermore, NHIRD contains the aforementioned catastrophic illness enrolment profile, and the data of these patients are compiled as the Registry for Catastrophic Illness Patient Database (RCIPD). In the present study, we enrolled lupus patients with detailed data in the RCIPD.

### Diagnosis of pneumocystis pneumonia

PCP was identified using the International Classification of Diseases (9th Revision) Clinical Modification (ICD-9-CM) codes for PCP (136.3) with the concurrent administration of medications used to treat PCP. Medications (Anatomical Therapeutic Chemical code) for PCP included Trimethoprim-sulfamethoxazole (J01EE01), Trimethoprim (J01EA01), Sulfamethoxazole (J01EC01), Clindamycin (J01FF01), Caspofungin (J02AX04), Micafungin (J02AX05), and Anidulafungin (J02AX06) [9].

### Matched non-PCP lupus subjects

In this study, SLE patients were identified with having a hospital admission or ambulatory visit with a diagnosis of SLE (ICD-9-CM code 710.0) and the catastrophic illness certificate of SLE between 1997 and 2013. The index date of PCP in lupus patients was defined as their first date of the visit with a PCP diagnosis, and their index year was defined as the year of index-date. We randomly selected matched non-PCP lupus subjects among those with SLE but without PCP, matching PCP cases (1:6) for age, gender, disease duration as well as the index year.

### Covariates

Covariates included in the multivariable conditional logistic regression model consisted of comorbidities and SLE-related medications within 3 months before the index date. Given that lupus nephritis has been implicated with an elevated risk for PCP in lupus patients, covariates regarding comorbid conditions included moderate to severe renal disease and the Charlson comorbidity index (CCI) calculated without inclusion of moderate to severe renal disease [10]. The comorbidity was identified as having one inpatient visit or at least three ambulatory visits with a corresponding ICD-9 CM code within one year prior to the defined index-date. The CCI was used to determine the comorbid medical conditions [11]. The main focus of this study was to investigate the risk for PCP infection with SLE relevant medications within 3 months before the index date. SLE-related medications included GC (average daily prednisolone equivalent dose ≤5 mg, 5–10 mg, > 10 mg), HCQ (not use, ≤ median 3-month cumulative dose, > median 3-month cumulative dose), SSZ, methotrexate (MTX), leflunomide, and immunosuppressants including cyclophosphamide (CYC) (not use, ≤ median 3-month cumulative dose, > median 3-month cumulative dose), cyclosporine (CS), azathioprine (AZA), and mycophenolate mofetil (MMF)/mycophenolic acid (MPA). Because trimethoprim-sulfamethoxazole was a well-known antibiotic that decreases the risk of PCP development, we also considered its use within 3 months before the index date as a covariate.

### Statistical analysis

Data were presented as numbers (percentages) for categorical variables and the mean ± standard deviation for continuous variables. The differences between the PJP group and non-PJP group were determined by Pearson’s *χ*^2^ test for categorical variables and Student’s t-test for continuous variables. Multivariable conditional logistic regression were applied to estimate the associations between covariates and the risk of PCP shown as odds ratio (OR) with 95% confidence intervals (CIs). We used forced entering and stepwise selection methods to build two models. Akaike information criterion (AIC) was used to evaluate the relative quality of statistical models [12]. In brief, AIC measures the relative amount of information decreased by a given model, and the low AIC hence reflects the statistical model with high quality. We assessed the significance of the interaction between HCQ and other covariates on PCP risk by using the Wald test to determine the *P*-value of the coefficient associated with the product of the indicator of HCQ and each indicator of the covariate. All of the results were analysed using SAS version 9.3 (SAS Institute, Inc., Cary, NC, USA), and a *P*-value < 0.05 was considered statistically significant.

## Results

### Demographic data of the enrolled population

A total of 24,343 patients with newly-diagnosed SLE from 2001 to 2013 were enrolled. Of them, we identified 58 patients who developed PCP as PCP cases and selected 348 matched non-PCP patients as controls. We noted that PCP cases appeared to have a higher CCI (2.4 ± 1.6 vs. 1.4 ± 1.1, *p* < 0.01) and were more likely to have renal diseases (34.5% vs. 8.6%, p < 0.01) than non-PCP controls (Table 1) (see dataset for details). Compared with non-PCP controls, patients with PCP tended to receive GC (96.6% vs. 69.0%, *p* < 0.01), and the dosage of GC was higher (36.4 ± 33.9 mg/day vs. 8.2 ± 19.8 mg/day, *p* < 0.01). With regards to immunosuppressants, PCP cases were more likely to receive CYC (31.0% vs. 7.2%, p < 0.01), MMF (22.4% vs. 1.4%, p < 0.01), and CYS (17.2% vs. 3.7%, p < 0.01) than controls. Conversely, PCP cases were less likely to receive HCQ (37.9% vs. 54.3%, *p* = 0.02) compared with non-PCP controls. (See Additional file 1: supplemental dataset for details).

**Table 1:** Demographic data and medications among enrolled subjects

	Control	Case	*P* value
	*n* = 348	*n* = 58	
Age, years	40.4 ± 17.9	40.4 ± 18.0	1.00
Gender			
Female	264 (75.9)	44 (75.9)	1.00
Male	84 (24.1)	14 (24.1)	
Disease duration, mean ± SD year	5.7 ± 4.6	4.8 ± 4.3	0.16
Comorbidities within one year before the index date			
CCI without moderate to severe renal disease, mean ± SD	1.2 ± 0.8	1.7 ± 1.4	0.01
Moderate to severe renal disease	30 (8.6)	20 (34.5)	< 0.01
Medications within 3 months before the indiex date			
Trimethoprim-sulfamethaxazole	7 (2.0)	0 (0.0)	0.28
Glucocorticoids			
Usage of glucocorticoids	240 (69.0)	56 (96.6)	< 0.01
Prednisolone equivalent, mean ± SD mg/day^a^	8.2 ± 19.8	36.4 ± 33.9	< 0.01
≤ 5 mg/day	217 (62.4)	2 (3.4)	< 0.01
5–10 mg/day	70 (20.1)	7 (12.1)	
> 10 mg/day	61 (17.5)	49 (84.5)	
Hydroxychloroquine	189 (54.3)	22 (37.9)	0.02
Cumulative dose (g)	9.2 ± 11.6	7.2 ± 12.0	0.23
None	159 (45.7)	36 (62.1)	0.06
≤ 14 g^b^	94 (27.0)	12 (20.7)	
> 14 g^b^	95 (27.3)	10 (17.2)	
Sulfasalazine	7 (2.0)	1 (1.7)	0.88
Cyclophosphamide	25 (7.2)	18 (31)	< 0.01
Cumulative dose (g)	0.1 ± 0.5	0.9 ± 1.7	< 0.01
None	323 (92.8)	40 (69.0)	< 0.01
≤1.4 g^b^	17 (4.9)	6 (10.3)	
> 1.4 g^b^	8 (2.3)	12 (20.7)	
Methotrexate	15 (4.3)	2 (3.4)	0.76
Leflunomide	0 (0.0)	0 (0.0)	NA
Mycophenolate mofetil /mycophenolic acid	5 (1.4)	13 (22.4)	< 0.01
Cyclosporine	13 (3.7)	10 (17.2)	< 0.01
Azathioprine	77 (22.1)	13 (22.4)	0.96

Data were shown as number (percentage) unless specified otherwise

^a^Prednisolone equivalent. ^b^Median cumulative dose. Abbreviations: IQR, interquartile range; SD, standard deviation; CCI: Charlson comorbidity index; NA, not applicable

### The distinct risk for PCP among frequently administered medications in SLE

We then assessed the association of the risk for PCP with each medication through conducting multivariable conditional logistical regression analyses using two models with different covariate selection methods. Determinants for the risk of PCP were largely the same between the model that forcibly entered all covariates (AIC = 85) and the model that used a stepwise selection of covariates (AIC = 82). In the stepwise selection model, determinants of the risk of PCP included moderate to severe renal disease (OR 6.73, 95% CI 1.98–22.92) and the use of GC at a dose-response manner (≤5 mg/day, reference; 5–10 mg/day, OR 25.88, 95% CI 2.97–225.33; > 10 mg/day, OR 286.58, 95% CI 28.58–> 999). (Table 2) Furthermore, the development of PCP in lupus patients was positively associated the use of MMF/MPA (OR 50.79, 95% CI 5.32–484.77) and CYC with 3-month cumulative dose > 1.4 g (OR 11.52, 95% CI 1.97–67.39). To determine the dose-response effect of HCQ on the risk for PCP, the use of HCQ was further categorised according to the median 3-month cumulative dose. Use of HCQ with 3-month cumulative dose > 14 g decreased the risk of PCP (OR 0.20, 95% CI 0.05–0.71), and use of HCQ with 3-month cumulative dose ≤14 g tended to decrease PCP risk (aOR 0.69, 95% CI 0.21–2.24). Collectively, these data demonstrated the distinct risks for PCP among medications used to treat SLE, and we found that moderate to severe renal disease as well as the use of GC, high dose CYC and MMF were major risk factors for PCP, whereas use of HCQ tended to be a protective factor for PCP in lupus patients.

**Table 2:** Crude and adjusted odds ratios for the association between variables and the risk of Pneumocystis Pneumonia

	Univariable analysis		Multivariable analysis			
			Forced entering		Stepwise selection	
	OR (95% CI)	P-value	OR (95% CI)	P-value	OR (95% CI)	P-value
Disease duration, incremental year	0.94 (0.88–1.01)	0.10	1.10 (0.92–1.32)	0.31		
CCI without moderate to severe renal disease	1.65 (1.23–2.22)	< 0.01	1.51 (0.91–2.49)	0.12		
Moderate to severe renal disease	4.97 (2.62–9.42)	< 0.01	7.61 (1.80–32.16)	< 0.01	6.73 (1.98–22.92)	< 0.01
Trimethoprim-sulfamethaxazole	< 0.001 (< 0.001–> 999)	0.99	< 0.001 (< 0.001–> 999)	0.99		
Glucocorticoids^a^						
≤ 5 mg/day	Ref.		Ref.		Ref.	
5–10 mg/day	8.76 (1.75–43.81)	< 0.01	23.73 (2.18–258.63)	< 0.01	25.88 (2.97–225.33)	< 0.01
> 10 mg/day	90.63 (20.54–399.95)	< 0.01	997.90 (51.32–> 999)	< 0.01	286.58 (28.58–> 999)	< 0.01
Hydroxychloroquine cumulative dose						
None	Ref.		Ref.		Ref.	
≤ 14 g^b^	0.56 (0.28–1.14)	0.11	0.56 (0.14–2.26)	0.41	0.69 (0.21–2.24)	0.54
> 14 g^b^	0.45 (0.21–0.97)	0.04	0.12 (0.02–0.63)	0.01	0.20 (0.05–0.71)	0.01
Sulfasalazine	0.85 (0.10–7.20)	0.88	0.08 (0.00–4.71)	0.22		
Cyclophosphamide cumulative dose						
None	Ref.		Ref.		Ref.	
≤ 1.4 g^b^	3.18 (1.17–8.62)	0.02	0.42 (0.08–2.15)	0.30	0.64 (0.14–3.01)	0.58
> 1.4 g^b^	21.87 (6.00–79.81)	< 0.01	12.01 (1.42–101.58)	0.02	11.52 (1.97–67.39)	< 0.01
Methotrexate / Leflunomide	0.79 (0.17–3.62)	0.76	0.29 (0.01–7.94)	0.47		
Mycophenolate mofetil/mycophenolic acid	34.11 (7.65–152.19)	< 0.01	38.07 (0.88–> 999)	0.06	50.79 (5.32–484.77)	< 0.01
Cyclosporine	5.03 (2.12–11.92)	< 0.01	3.83 (0.64–23.05)	0.14		
Azathioprine	1.02 (0.53–1.96)	0.96	0.57 (0.15–2.26)	0.43		
AIC			85		82	

Adjusted for age and gender. ^a^Prednisolone equivalent. ^b^Median cumulative dose. AIC, Akaike information criterion

### Subgroup analyses of the association between the risk for PCP and HCQ usage

Subgroup analyses were conducted to test the consistency of the association between HCQ and PCP risk and to examine the presence of interaction effect between HCQ and other relevant variables. We further divided the subjects by age (> 40 and ≤ 40 years), sex, moderate to severe renal disease, and the usage of immunosuppressants (CYC, MMF/MPA, CS or AZA) (Table 3). No interaction effect was noted, and the protective effect of HCQ higher than median dose against PCP appeared to be prominent in those with young age (≤40 years), were female, without renal diseases and receiving GC higher than 10 mg/day. Collectively, these data demonstrated a consistently inverse correlation between HCQ and the risk PCP in patients with SLE.

**Table 3:** Subgroup analyses for the correlation between hydroxychloroquine and risk of pneumocystis pneumonia using multivariable conditional logistic regression models

Groups	None	Hydroxychloroquine 3-month cumulative dose				
		≤14 g^a^		> 14 g^a^		
		OR (95% CI)	P-value	OR (95% CI)	P-value	
Age						0.052
Age ≤ 40 years	Ref.	0.89 (0.26–3.07)	0.85	0.10 (0.02–0.54)	0.01	
Age > 40 years	Ref.	0.24 (0.04–1.31)	0.10	0.60 (0.15–2.49)	0.49	
Gender						0.42
Female	Ref.	0.74 (0.27–2.07)	0.57	0.26 (0.08–0.80)	0.02	
Male	Ref.	0.13 (0.01–1.94)	0.14	0.55 (0.02–16.59)	0.73	
Moderate to severe renal disease						0.41
No	Ref.	0.35 (0.11–1.12)	0.08	0.22 (0.07–0.73)	0.01	
Yes	Ref.	0.71 (0.05–11.11)	0.80	0.03 (< 0.001–1.19)	0.06	
Glucocorticoids^b^						0.31
≤10 mg/day	Ref.	0.25 (0.05–1.38)	0.11	0.50 (0.10–2.41)	0.39	
> 10 mg/day	Ref.	0.95 (0.27–3.35)	0.94	0.17 (0.04–0.68)	0.01	
Immunosuppressants^c^						0.83
No	Ref.	< 0.001 (< 0.001–> 999)	0.94	0.09 (0.00–1.67)	0.11	
Yes	Ref.	1.09 (0.37–3.19)	0.88	0.39 (0.13–1.20)	0.10	

Covariates in the conditional logistric regression models included Charlson comorbidity index without moderate to renal disease, moderate to severe renal disease, methotrexate/leflunomide, sulfasalazine, immunosuppressants and trimethoprim-sulfamethaxazole excluding the covariate that was used to stratify subjects. ^a^Median cumulative dose. ^b^Prednisolone equivalent. ^c^Immunosuppressants include cyclosporine, azathioprine, cyclophosphamide, and mycophenolate mofetil/mycophenolic acid

## Discussion

To the best of our knowledge, the present study is the first research to address the association between HCQ use and the risk for PCP in lupus patients using a population-based claim database. We found that the use of HCQ with a 3-month cumulative dose of > 14 g was associated with a reduced risk of PCP, whereas the use of GC, CYC and MMF/MPA was associated with an increased risk for PCP in lupus patients. These findings indicated the distinct risk for PCP in lupus patients receiving GC and immunosuppressants and suggested a potential protective role of HCQ against PCP.

HCQ was originally developed as an antimalarial medication and is currently widely used as an immunomodulation agent for autoimmune diseases, mainly SLE, RA and Sjögren’s syndrome [13]. In addition to the antimalarial properties, HCQ also has antibacterial activities, which were mainly exerted by pH-dependent iron depletion and by an increased pH of phagosome, which in turn inhibit the growth of intracellular organisms [4]. Owing to the aforementioned antibacterial effect, HCQ has been found to be associated with a decreased risk of major bacterial infections in lupus patients [14, 15]. In addition to the antimalarial and antibacterial effects, HCQ also exhibits antifungal activities. Henriet et al., conducting an in vivo study with *Aspergillus fumigatus and Aspergillus nidulans*, demonstrated that chloroquine may increase the antifungal activity of leucocytes isolated in those with chronic granulomatous disease (CGD) at a low concentration through regulating the production of interleukin-1β and tumor necrosis factor-α [16]. Another in vitro study also found the synergistic effect of chloroquine and fluconazole against fluconazole-resistant *Candida spp*. including *Candida tropicalis* as well as *Candida krusei* [5]. Although data regarding *Pneumocystis jirovecii* are still lacking, Podrebarac et al. reported two subjects with SLE receiving high-dose GC developed PCP shortly after discontinuation of HCQ [17]. Taken together, these evidence highlight the anti-fungal effect of HCQ and at least partly explain the protective effect of HCQ against PCP in lupus patients as shown in this study.

The prevalence of PCP in lupus patients has varied over the past two decades. Gupta D. et al., analyzing 18 studies conducted in the United States between 1987 and 2006, found that merely 0.16% (121/76,156) of patients with SLE had PCP [18]. Recently, Kapoor TM et al. reported that approximately 0.45% (9/2000) of hospitalized patients with SLE had PCP using data collected from a database for the period 2000–2014 at Columbia University Medical Center-New York Presbyterian Hospital [19]. In the present study, we found that 0.24% (58/24,348) of patients with SLE between 2001 and 2013 had PCP in Taiwan. In line with our data, Weng CT et al., investigating 858 hospitalised lupus patient in southern Taiwan, found that only 0.58% (5/858) of them had PCP with identified Pneumocystis organisms in the lung (2 by lung biopsy and the other three by bronchoalveolar lavage) [20]. Taken together, the diagnosis of PCP appears to have increased gradually in the past two decades; however, the prevalence remains low. Given that we used a conservative definition for PCP, including both ICD-9-CM codes of PCP and medication for PCP to define the diagnosis of PCP, we may have underestimated, rather than overestimated, the prevalence of PCP infection in the present Taiwanese research.

In the present study, we identified an elevated risk of PCP in lupus patients taking MMF/MPA or in taking CYC with 3-month cumulative dose > 1.4 g. However, the use of CS, AZA or MTX was not significantly associated with the risk for PCP. Indeed, discrepant findings have been noted in the risk for infection among immunosuppressants in lupus patients [3, 21]. MMF was initially reported to have fewer severe infections as the induction therapy for lupus nephritis when compared with CYC [22, 23]. However, one recently published study conducted by Feldman CH et al., using the Medicaid beneficiaries with patients with SLE in 28 U.S. states, found that the risk for severe infections was not increased among new users of CYC and MMF/MPA, and AZA [21]. Similarly, Mok CC et al. conducted a long-term follow-up research among 803 patients with SLE and showed that CYC and MMF/MPA had similar impacts on survival [24]. However, it is somehow difficult to disentangle the effects of the complex interaction among disease activities, the sequential/combinational usage of immunosuppressants, and dosage of each medication on the risks for PCP. Therefore, CS and AZA did not confer any significant independent risks for PCP in this study, although these two medications tended to increase the risk of PCP in the univariable analysis. Intriguingly, in the aforementioned study conducted by Mok CC et al., the use of HCQ had a survival benefit in patients with SLE (HR 0.59, 95% CI 0.37–0.93) [24]. Moreover, Feldman CH et al., using Medicaid database between 2000 and 2006, also found that HCQ user had a decreased risk of infections than non-user (HR 0.73, 95% CI 0.68–0.77) [10], and these findings are largely consistent with our findings in the present study.

Moderate to severe renal disease was an independent risk for PCP in the present study. In line with our findings, Feldman CH et al., analyzing 33,565 patients with SLE and 7113 with lupus nephritis, found that the incidence rate of serious infections requiring hospitalization per 100 person-years was 10.8 in SLE and 23.9 in those with lupus nephritis [10]. Although we could not specify lupus nephritis using ICD, the moderate to severe renal disease in lupus patients should be reasonably attributed to lupus nephritis given that underlying moderate to severe renal disease is uncommon in subjects aged 40. The high risk for PCP in lupus patients with moderate to severe renal disease highlights that lupus patients with renal involvement are particularly vulnerable to infectious diseases, including PCP. A number of studies have explored the efficacy and safety profile of using trimethoprim-sulfamethoxazole as PCP prophylaxis in high-risk lupus patients, particularly those receiving medium-to-high dose GC [25, 26]. However, there is currently no consensus regarding routine PCP prophylaxis in lupus patients, and we think PCP prophylaxis might potentially be feasible in high-risk patients [18].

There are limitations in this study. First, our results were derived from a population-based claim database in Taiwan; therefore, further investigations involving other populations are needed for the generalizability. Second, we could not assess the causality of HCQ-associated reduced risk for PCP; however, the finding of this study warrant more mechanistic studies. Third, the accuracy of diagnoses is a concern in the claim database. However, we think that regular quality control surveys of NHIRD among all medical facilities by the BNHI has much improved the accuracy of coding in NHIRD [27], and bias attributed by the misclassification was hence minimised. Additionally, the prevalence of PCP infection in the present Taiwanese research was quite similar to those in other studies [19, 20].

## Conclusions

PCP is a life-threatening fungal infection with an increasing prevalence in patients with SLE. We have identified a potentially protective effect of HCQ against PCP and demonstrated the distinct risks for PCP conferred by GC and various immunosuppressants in lupus patients. The decreased risk of PCP in lupus patients receiving HCQ as we showed should be clinically relevant, such as the prescription of HCQ for lupus patients with a high risk for PCP and the less indicated for PCP prophylactics in patients already receiving HCQ. The distinct risks for PCP among GC and immunosuppressants should be taken into account with regards to the vigilance for PCP and the implementation of PCP prophylaxis in lupus patients. Further mechanistic studies are required to address the underlying mechanisms.

## Supplementary information

**Additional file 1.** Dataset. raw data of the study

## Data Availability

All of the data in the present research are contained in the article and the supplementary profile.

## Abbreviations

AZA: Azathioprine
BNHI: Bureau of National Health Insurance
CCI: Charlson comorbidity index
CI: Confidence intervals
CS: Cyclosporin
CYC: Cyclophosphamide
GC: Glucocorticoids
HCQ: Hydroxychloroquine
ICD: International Classification of Diseases
MMF: Mycophenolate mofetil
MPA: Mycophenolic acid
NHI: National Health Insurance
NHIRD: National Health Insurance Research Database
NSAID: Non-steroid anti-inflammatory drugs
OR: Odds ratios
PCP: Pneumocystis pneumonia
RA: Rheumatoid arthritis
RCIPD: Registry for Catastrophic Illness Patient Database
SLE: Systemic lupus erythematosus
SSZ: Sulfasalazine

## References

[1] Fillatre P, Decaux O, Jouneau S, Revest M, Gacouin A, Robert-Gangneux F, Fresnel A, Guiguen C, Le Tulzo Y, Jego P (2014). Incidence of Pneumocystis jiroveci pneumonia among groups at risk in HIV-negative patients. Am J Med.

[2] Schmidt JJ, Lueck C, Ziesing S, Stoll M, Haller H, Gottlieb J, Eder M, Welte T, Hoeper MM, Scherag A (2018). Clinical course, treatment and outcome of Pneumocystis pneumonia in immunocompromised adults: a retrospective analysis over 17 years. Crit Care.

[3] Pryor BD, Bologna SG, Kahl LE (1996). Risk factors for serious infection during treatment with cyclophosphamide and high-dose corticosteroids for systemic lupus erythematosus. Arthritis Rheum.

[4] Rolain JM, Colson P, Raoult D (2007). Recycling of chloroquine and its hydroxyl analogue to face bacterial, fungal and viral infections in the 21st century. Int J Antimicrob Agents.

[5] Li Y, Wan Z, Liu W, Li R (2015). Synergistic activity of chloroquine with fluconazole against fluconazole-resistant isolates of Candida species. Antimicrob Agents Chemother.

[6] Wang J, Gigliotti F, Bhagwat SP, George TC, Wright TW (2010). Immune modulation with sulfasalazine attenuates immunopathogenesis but enhances macrophage-mediated fungal clearance during Pneumocystis pneumonia. PLoS Pathog.

[7] Nunokawa T, Yokogawa N, Shimada K, Sugii S, Nishino J, Gosho M, Wagatsuma Y, Tohma S (2019). Prophylactic effect of sulfasalazine against Pneumocystis pneumonia in patients with rheumatoid arthritis: a nested case-control study. Semin Arthritis Rheum.

[8] The National Health Insurance Statistics 2015. In*.*: National Health Insurance Administration, Ministry of Health and Welfare, Taiwan, R.O.C.; 2015.

[9] Maschmeyer G, Helweg-Larsen J, Pagano L, Robin C, Cordonnier C, Schellongowski P, The European conference on infections in leukemia ajvoTEGfB, marrow transplantation TEOfR, treatment of Cancer tIIHS, the European L (2016). ECIL guidelines for treatment of Pneumocystis jirovecii pneumonia in non-HIV-infected haematology patients. J Antimicrob Chemother.

[10] Feldman CH, Hiraki LT, Winkelmayer WC, Marty FM, Franklin JM, Kim SC, Costenbader KH (2015). Serious infections among adult Medicaid beneficiaries with systemic lupus erythematosus and lupus nephritis. Arthritis Rheumatol.

[11] Deyo RA, Cherkin DC, Ciol MA (1992). Adapting a clinical comorbidity index for use with ICD-9-CM administrative databases. J Clin Epidemiol.

[12] Vrieze SI (2012). Model selection and psychological theory: a discussion of the differences between the Akaike information criterion (AIC) and the Bayesian information criterion (BIC). Psychol Methods.

[13] Avina-Zubieta JA, Galindo-Rodriguez G, Newman S, Suarez-Almazor ME, Russell AS (1998). Long-term effectiveness of antimalarial drugs in rheumatic diseases. Ann Rheum Dis.

[14] Ruiz-Irastorza G, Olivares N, Ruiz-Arruza I, Martinez-Berriotxoa A, Egurbide MV, Aguirre C (2009). Predictors of major infections in systemic lupus erythematosus. Arthritis Res Ther.

[15] Siso A, Ramos-Casals M, Bove A, Brito-Zeron P, Soria N, Munoz S, Testi A, Plaza J, Sentis J, Coca A (2008). Previous antimalarial therapy in patients diagnosed with lupus nephritis: influence on outcomes and survival. Lupus.

[16] Henriet SS, Jans J, Simonetti E, Kwon-Chung KJ, Rijs AJ, Hermans PW, Holland SM, de Jonge MI, Warris A (2013). Chloroquine modulates the fungal immune response in phagocytic cells from patients with chronic granulomatous disease. J Infect Dis.

[17] Podrebarac TA, Jovaisas A, Karsh J (1996). Pneumocystis carinii pneumonia after discontinuation of hydroxychloroquine in 2 patients with systemic lupus erythematosus. J Rheumatol.

[18] Gupta D, Zachariah A, Roppelt H, Patel AM, Gruber BL (2008). Prophylactic antibiotic usage for Pneumocystis jirovecii pneumonia in patients with systemic lupus erythematosus on cyclophosphamide: a survey of US rheumatologists and the review of literature. J Clin Rheumatol.

[19] Kapoor TM, Mahadeshwar P, Nguyen S, Li J, Kapoor S, Bathon J, Giles J, Askanase A (2017). Low prevalence of Pneumocystis pneumonia in hospitalized patients with systemic lupus erythematosus: review of a clinical data warehouse. Lupus.

[20] Weng CT, Liu MF, Weng MY, Lee NY, Wang MC, Lin WC, Ou CY, Lai WW, Hsu SC, Chao SC (2013). Pneumocystis jirovecii pneumonia in systemic lupus erythematosus from southern Taiwan. J Clin Rheumatol.

[21] Feldman CH, Marty FM, Winkelmayer WC, Guan H, Franklin JM, Solomon DH, Costenbader KH, Kim SC (2017). Comparative rates of serious infections among patients with systemic lupus Erythematosus receiving immunosuppressive medications. Arthritis Rheumatol.

[22] Ginzler EM, Dooley MA, Aranow C, Kim MY, Buyon J, Merrill JT, Petri M, Gilkeson GS, Wallace DJ, Weisman MH (2005). Mycophenolate mofetil or intravenous cyclophosphamide for lupus nephritis. N Engl J Med.

[23] Liu LL, Jiang Y, Wang LN, Yao L, Li ZL (2012). Efficacy and safety of mycophenolate mofetil versus cyclophosphamide for induction therapy of lupus nephritis: a meta-analysis of randomized controlled trials. Drugs.

[24] Mok CC, Tse SM, Chan KL, Ho LY (2018). Effect of immunosuppressive therapies on survival of systemic lupus erythematosus: a propensity score analysis of a longitudinal cohort. Lupus.

[25] Park JW, Curtis JR, Moon J, Song YW, Kim S, Lee EB (2018). Prophylactic effect of trimethoprim-sulfamethoxazole for pneumocystis pneumonia in patients with rheumatic diseases exposed to prolonged high-dose glucocorticoids. Ann Rheum Dis.

[26] Park JW, Curtis JR, Kim MJ, Lee H, Song YW, Lee EB (2019). Pneumocystis pneumonia in patients with rheumatic diseases receiving prolonged, non-high-dose steroids-clinical implication of primary prophylaxis using trimethoprim-sulfamethoxazole. Arthritis Res Ther.

[27] Cheng TM (2003). Taiwan’s new national health insurance program: genesis and experience so far. Health Aff (Millwood).

